# Brain image data processing using collaborative data workflows on Texera

**DOI:** 10.3389/fncir.2024.1398884

**Published:** 2024-07-10

**Authors:** Yunyan Ding, Yicong Huang, Pan Gao, Andy Thai, Atchuth Naveen Chilaparasetti, M. Gopi, Xiangmin Xu, Chen Li

**Affiliations:** ^1^Department of Computer Science, University of California, Irvine, Irvine, CA, United States; ^2^Department of Anatomy and Neurobiology, School of Medicine, University of California, Irvine, Irvine, CA, United States; ^3^Department of Biomedical Engineering, University of California, Irvine, Irvine, CA, United States; ^4^The Center for Neural Circuit Mapping, University of California, Irvine, Irvine, CA, United States

**Keywords:** TissueCyte, circuit tracing, mouse brain, data analytics, image stitching, 3D visualization

## Abstract

In the realm of neuroscience, mapping the three-dimensional (3D) neural circuitry and architecture of the brain is important for advancing our understanding of neural circuit organization and function. This study presents a novel pipeline that transforms mouse brain samples into detailed 3D brain models using a collaborative data analytics platform called “Texera.” The user-friendly Texera platform allows for effective interdisciplinary collaboration between team members in neuroscience, computer vision, and data processing. Our pipeline utilizes the tile images from a serial two-photon tomography/TissueCyte system, then stitches tile images into brain section images, and constructs 3D whole-brain image datasets. The resulting 3D data supports downstream analyses, including 3D whole-brain registration, atlas-based segmentation, cell counting, and high-resolution volumetric visualization. Using this platform, we implemented specialized optimization methods and obtained significant performance enhancement in workflow operations. We expect the neuroscience community can adopt our approach for large-scale image-based data processing and analysis.

## 1 Introduction

In neuroscience, large high-resolution imaged brain data is frequently needed to facilitate accurate neural circuit mapping and subsequent image analysis (Oh et al., 2014; Kim et al., 2015, 2017; Liebmann et al., 2016; Mano et al., 2018; Whitesell et al., 2019; Zhang et al., 2023). Gathering accurate, high-resolution data requires sectioning methods and additional post-processing to correct flaws from imaging. Traditional histological methods for imaging the brain in 3D rely on manually sectioning of the whole brain, followed by the mounting and scanning of all sections. These processes are not only labor-intensive but also prone to errors, limiting their effectiveness (Stille et al., 2013). Alternative procedures, such as tissue clearing (Dodt et al., 2007; Chung et al., 2013; Renier et al., 2014; Jing et al., 2018; Susaki et al., 2020; Ueda et al., 2020; Kosmidis et al., 2021) combined with light-sheet microscopy, offer a different approach but often result in deformed samples, outputting overly expanded or contracted images. These methods can complicate subsequent registration processes due to issues with potential chemical-induced fluorescence quenching or incomplete clearing.

The recent emergence of sectioning-based 3D reconstruction techniques, including Serial Two-Photon Tomography (STPT/TissueCyte) (Ragan et al., 2012; Osten and Margrie, 2013; Kim et al., 2015), marks a significant step forward. These techniques automate the imaging and sectioning processes and mitigate the challenges associated with manual sectioning, such as image misalignments and registration inaccuracies. Despite these advances, platforms such as TissueCyte still require extensive post-imaging processing to achieve accurate 3D reconstructions.

The construction of 3D mouse brain data requires the acquisition of high-resolution 2D images of brain sections. Each section is imaged in segments, or tiles, which are then pieced together to form a complete 2D representation of the section. Compiling a sufficient number of these 2D composite sections and stacking them accurately is essential for recreating the 3D architecture of a mouse brain. The quality of the initial images and their proper sectional alignment collectively contribute to the fidelity of the 3D data.

**Figure 1:**
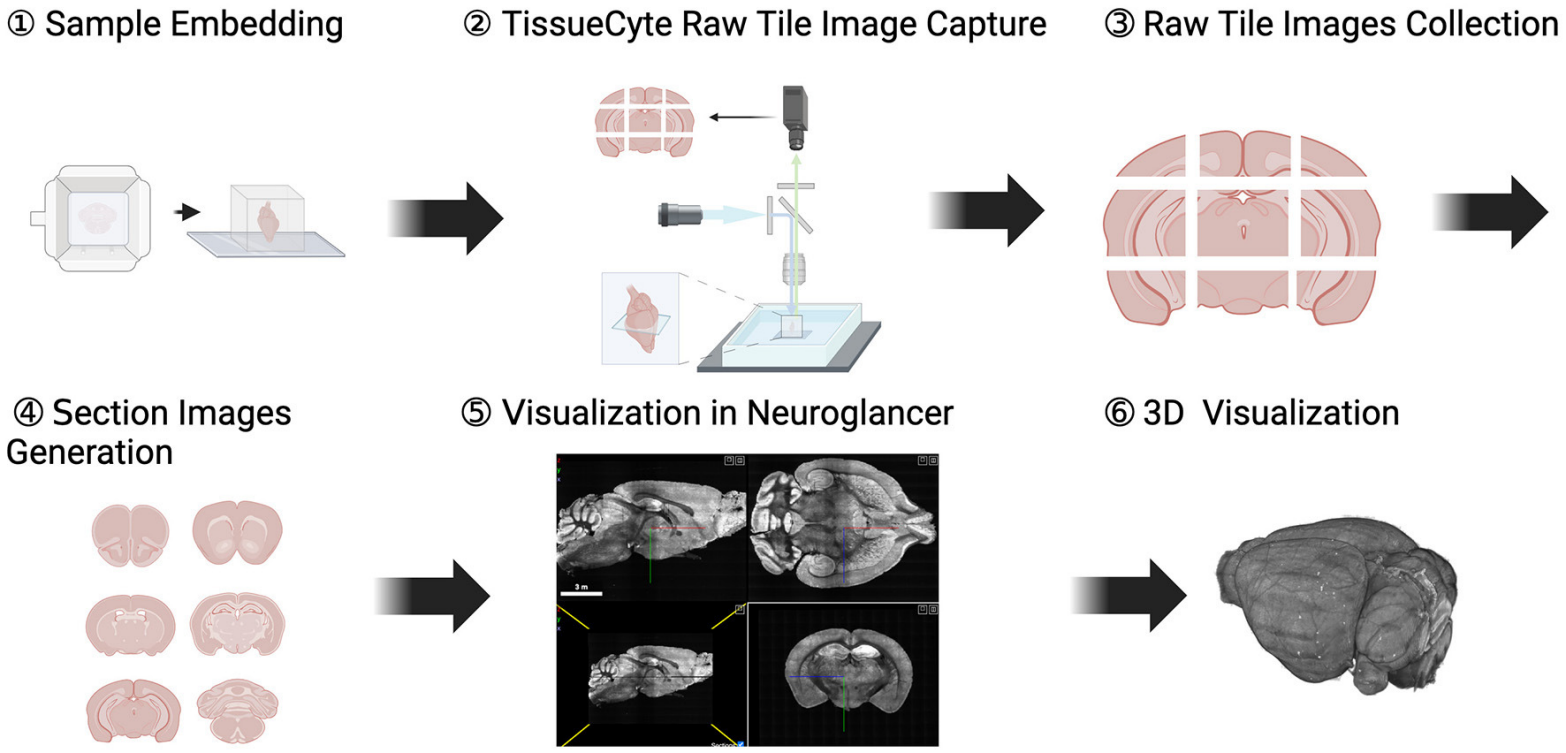
Overview of a process from a mouse brain to final digitized images and visualizations. This process includes comprehensive steps involved in converting physical brain samples to detailed 2D section images and finally to a 3D volume for visualization and analysis.

In this study, we introduce a pipeline tailored for transforming mouse brain samples to detailed whole brain volumes. Our pipeline involves capturing high-resolution images via TissueCyte, efficiently and accurately stitching the tile images, and stacking 2D sections to construct a detailed whole mouse brain volume. This pipeline was developed by experts in three distinct scientific disciplines: neuroscience, computer vision, and data systems. An overview of our comprehensive process is provided in Figure 1. Initially, mouse brain samples are embedded in agarose and subsequently imaged using TissueCyte microscopy to obtain high-resolution, multichannel tile images. For a typical whole mouse brain, this methodology yields 616 tile images across four channels, with each image being 1.4 MB in size with a resolution of 832 × 832 pixels. Each section image, which contains 8, 716 × 11, 236 pixels, is subject to deformation correction for each tile to enable precise stitching. Uniform intensity values across the entire image are also ensured through a brightness normalization process. Collectively, these measures allow for clear visualization of individually labeled cells, laying the foundation for subsequent analysis.

Due to the interdisciplinary nature of the study, we involve team members with diverse expertise and backgrounds. To ensure effective collaboration, we utilize a user-friendly platform that accommodates these diverse backgrounds, including those without programming skills. To this end, we use Texera (Texera, 2024) as a platform for efficient collaborations that satisfy these requirements. By using designated optimization methods on Texera, we complete the entire image processing in 2.5 h for a whole brain spanning 280 sections. This runtime performance achieves more than an 80% time reduction compared to the non-optimized approach and around a 30% time reduction compared to traditional parallel approaches. After stitching and outputting all section images, we use Neuroglancer (Maitin-Shepard et al., 2021), a web-based tool for volumetric data visualization, to render the brain data in 3D. The resulting 3D data is then ready for further analysis, including registration, annotation, and cell counting.

Our pipeline produces high-fidelity, seamless, and high-resolution 3D reconstructions of mouse brains, enabling intricate visualizations of pivotal biological markers throughout the brain structure. This precision comes from the accurate stitching of 2D brain sections, which allows for clear delineation and tracking of anatomical features, including cell somas, cell processes, and vascular networks in both 2D and 3D spaces. Furthermore, produced 3D data supports advanced procedures such as whole-brain 3D registration and subsequent atlas-based segmentation with the Allen Brain Atlas Common Coordinate Framework (CCF) (Wang et al., 2020). This data enables precise identification and quantification of region-specific signals, providing a robust platform for comprehensive neurobiological research.

The rest of the paper is organized as follows. We first describe our methodology for acquiring, managing, and processing image data in Section 2, illustrating the steps taken to prepare high-quality images for subsequent visualization and analysis. Next, we present the implementation of the methods, optimization strategies, and results in Section 3. We conclude with a discussion in Section 4.

## 2 Materials and methods

### 2.1 Animals and ethics

All experiments were conducted according to the National Institutes of Health guidelines for animal care and use, and were approved by the University of California, Irvine Institutional Animal Care and Use Committee (IACUC, protocol #: AUP-22-163) and the Institutional Biosafety Committee (IBC). Dataset B0039 was generated using a 2-month-old wild-type C57BL/6J mouse. Similarly, a 2-month-old Tie2-Cre; Ai9 mouse was used to produce the B0003 dataset.

### 2.2 Data acquisition

#### 2.2.1 Viral injections

The B0039 mouse received intracranial injections of viral tracers using a stereotaxic machine. The injections comprised a combination of Adeno-associated virus (AAV) helpers (pENN.AAV.CamKII 0.4.Cre.SV40, Addgene viral prep #105558-AAV1, 5.3 × 10^13^ GC/ml + AAV8-DIO-TC66T-2A-eGFP-2A-oG, Salk Institute, CA, USA, 2.36 × 10^13^ GC/ml) and engineered pseudo-typed rabies virus (EnvA-RV-SADΔG-DsRed, CNCM, 2.1 × 10^9^ IU/ml). The injection precisely labeled specific cell types in the brain region of interest, along with their monosynaptically connected input cells throughout the brain. Following an incubation period of 30 days, which included three weeks of AAV helper injections and nine days of engineered pseudo-typed rabies virus injections, transcardial perfusion was performed on mice using 1 × PBS followed by 4% PFA. Post-dissection, the mouse brains were fixed overnight at 4°*C* in 4% PFA to ensure the fixation of fluorescent-labeled cells.

#### 2.2.2 Brain sample preparation and TissueCyte imaging

In our study, we used a sample embedding method analogous to the one previously published (Oh et al., 2014). A brief description of the method is provided below for clarity and reproducibility. The mouse brains were transferred to a 1 × PB solution with 0.01% sodium azide after overnight PFA fixation until they were ready for imaging. The next step involved embedding the mouse brains in a 4% oxidized agarose solution, followed by immersion in a solution of Surecast (Acrylamide:Bis-acrylamide ratio of 29:1), with a total concentration of 4.5% Surecast and 0.5% VA-044 activator, with excess volume (>20 *ml* for a brain), diluted in 1 × PB: (22 ml PB; 3 ml Surecast; 0.13 g activator per brain) at 4°C overnight. The next day, the agarose-embedded brain was removed from the solution and placed into a disposable mold. It was then baked at 40°C for 2 h. Following the baking process, the agarose-embedded brain was transferred back to 1 × PB and allowed to soak overnight. Subsequently, the specimen was ready for TissueCyte imaging at 4°C.

**Figure 2:**
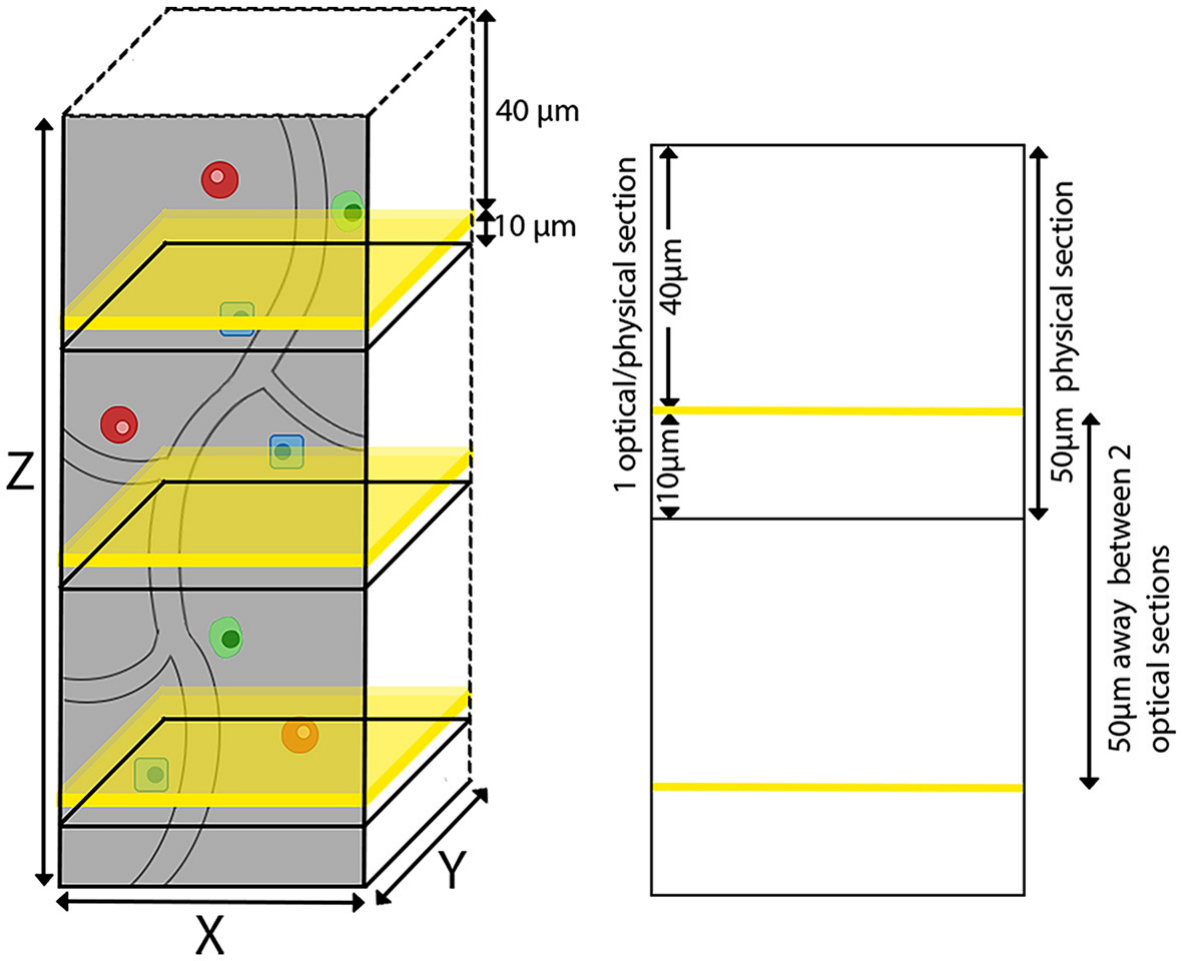
TissueCyte imaging setup utilizing both physical and optical sectioning. This illustration shows our imaging process, where each physical brain section of 50 μm in thickness is paired with a single optical section. The optical sections are represented by yellow planes located 40 μm below the surface.

The agarose-embedded brain was secured on a glass slide using adhesive, with magnets on the opposite side adhering to a metal plate at the base of the TissueCyte sample container filled with 1 × PB solution. In our specific setup, the mouse brain was affixed with the cerebellum facing upward and the olfactory bulb facing downward. TissueCyte, an automated block-face imaging technique, employs serial two-photon tomography (STPT) imaging modality to capture repetitive images through two-photon illumination while physically sectioning the imaged area with an integrated vibratome. The imaging setup employed specific parameters such as laser wavelength, laser power, resolution of each tile, number of tiles, and number of optical sections per cycle. TissueCyte employs a 16 × objective with a field of view (FOV) of 1,125 μm × 1,125 μm. Before imaging, all agarose was sectioned until the cerebellum was exposed. We then imaged one optical section situated 40 μm below the surface. This optical section comprised ~11 × 14 tiles for each color channel (red, green, blue, and far-red) for a regular whole mouse brain. After we completed the imaging for one optical section, the top surface was cut off by the integrated vibratome at a thickness of 50 μm, marking the completion of one cycle (Figure 2). The entire imaging process consists of 280–300 cycles, which can image the whole mouse brain in the coronal direction. The tile images collected for each single optical section were saved in the TIFF format, with a resolution of 832 × 832 pixels in the XY plane for each tile. Our imaging platform exhibits robustness and is well-aligned between sections, eliminating the need for additional alignment processing.

### 2.3 Image data and management

The image data were acquired by the TissueCyte 1600FC. For each entire brain, the TissueCyte 1600FC captured a substantial amount of image data. In our dataset, a whole brain was represented as 280 optical sections, where each section corresponded to a plane of the brain. Each section was typically made up of 154 (11 × 14) tiles, with each tile representing a specific segment of the section. Each segment or tile location had four files corresponding to four color channels: red, green, blue, and far-red, resulting in a total of 616 tile files. In total, each brain contained 689,920 tile images. Each tile image was ~1.4 MB, resulting in a total of 224 GB per brain volume.

We managed and structured the data files as follows. Two types of metadata files were stored on disk: brain volume metadata and section metadata. The tile images were stored in a hierarchy of folders. In each brain file's folder, we created a text file for the brain volume metadata and a subfolder for each brain section. Each section folder contained its tile images across the four channels, stored in the TIFF format. Furthermore, in each section folder, we stored metadata for the section, denoting information such as the number of tiles per row and column, the dimensions of the tiles, and other pertinent details. For instance, one of our brain datasets, labeled as B0039, had 280 section folders, each containing 616 tile images and a text file consisting of section metadata.

### 2.4 Image data processing

**Figure 3:**
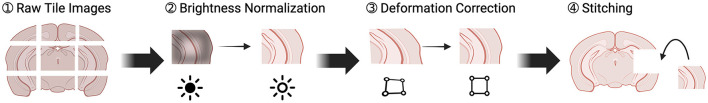
Pipeline of transforming individual tile images to section images. There were four steps. (1) Raw tiles were retrieved from the TissueCyte. (2) These raw tiles underwent brightness normalization to correct intensity values. (3) Lens distortion was corrected. (4) The tiles were stitched together to create a full section.

Our image processing aimed to create a comprehensive 3D brain data from tile images. As outlined in Figure 3, this process began by generating an image for each brain section from the tile images, and then using these section images to assemble a 3D data. The quality of the final 3D data was significantly influenced by the quality of the 2D section images. Therefore, we implemented two optimization steps to enhance the quality of the tile images. In this section, we discuss the tile stitching process, the two optimization steps, and conclude with the conversion from 2D to 3D.

#### 2.4.1 Stitching tile images into a 2D section image

During the stitching phase, we merged individual tiles into a cohesive singular image, eliminating harsh lines or abrupt changes where the tiles meet. In the initial set of imaged tiles, overlaps between neighboring tiles during the imaging process were unavoidable. These overlaps occurred because the brain sample moved during imaging with a step size of 1,017 μm either horizontally or vertically, creating an overlap of ~10% (100 pixels) with the 1,125 μm TissueCyte field of view between neighboring tiles. This caused each raw tile to interact and overlap with its immediate neighbors, including the tiles on its left, right, above, and below. Solving this problem required proper tile positioning, which depended on the location of the tiles within the section (rows and columns), the scale of the tiles, and the degree of overlap between tiles. Traditionally, to manage these overlaps, researchers commonly cut extraneous portions out from the tiles to correct the scale in a process known as trimming. An alternative approach involves accurately positioning the tile images during the stitching process, allowing the subsequent tile to cover the overlapping area. In this way, we could achieve the same result without explicitly trimming the tiles. We solved this problem by computing translation parameters for each tile. This was done by calculating the overlapping area between adjacent tiles and determining how much of each tile image extended into its neighboring tiles. Each tile must then be placed in its designated position within its section to ensure coherence in the final output section.

For illustration purposes, let us first analyze a group of four tiles arranged in a 2 × 2 configuration. Within this group, we assessed the horizontal shift, which is between a left tile and its right neighbor, and the vertical shift, which is between a top tile and its bottom neighbor. We selected specific pixel strips from each tile. From a left tile, we took a 100-pixel wide strip from its right edge, and from its right tile, we took a 50-pixel wide strip from its left edge. We aligned the narrower strip from the right tile to the broader strip of the left tile using normalized cross correlation. From this operation, we determined the number of pixels needed for each strip to be displaced so that the right tile aligned with the left precisely. The distance of this shift is defined as the translation parameters. As these displacements are consistent and uniform across sections and different brain scans, this process only needs to be performed once and the resulting parameters can be used across multiple scanning procedures. After obtaining the translation parameters, we can determine the precise position of each tile within the section pixel by pixel. We prepared a blank canvas corresponding to the section's dimensions. Each tile was then accurately positioned at its calculated location, measured in pixel units, based on the three previously mentioned factors. The values in overlapping regions were averaged together through a linear blending operation. Once all tiles were put in place, we obtained a high-resolution image of the entire section.

#### 2.4.2 Improving 2D image quality

Simply using the raw tiles collected by TissueCyte was insufficient, since environmental influences, lens-induced distortions, and image overlaps, originating from external factors, could significantly impact the quality of 3D visualization. To solve these problems, we proposed a series of pre-processing steps including brightness normalization and deformation corrections. These steps were designed to enhance the image quality and ensure a standardization of values across the datasets. In this section, we present these issues, outline the specific challenges we aim to address, and detail a solution for each of them.

##### 2.4.2.1 Brightness normalization

In our acquired tile images, their center regions typically exerted brighter values than the edges due to lens vignetting. When stitched together, the generated output did not have smooth brightness values along the overlapping edges.

As visualization remains a major goal of our framework, we aim to output clean and smooth visualizations. When presenting volumes in 3D, lens vignetting will introduce artifacts that result in a subpar visualization. This necessitates the correction of lens vignetting.

**Figure 4:**
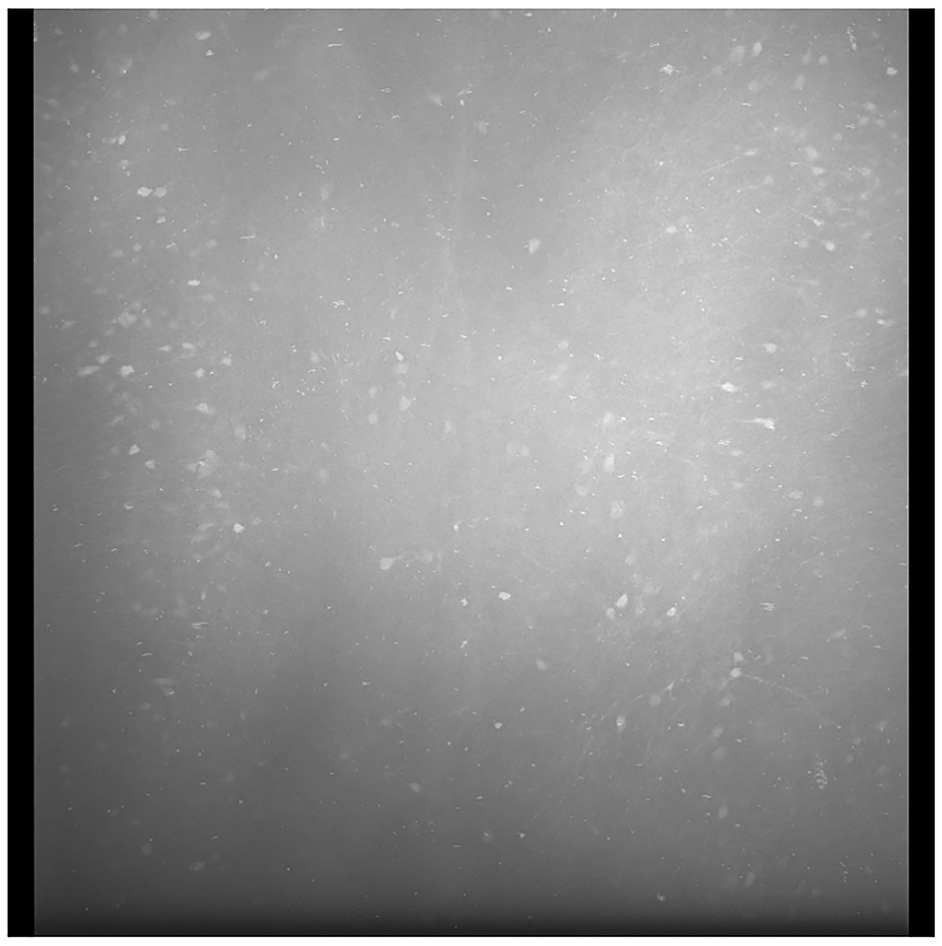
A sample average profile for a B0039 brain section. The edges are darker than the center regions of the image. Applying these average sections to each tile standardizes the brightness values across the entire stitched section and minimizes spots that are very bright or dark, as seen in the output depicted in Figure 5.

To solve this problem, we generated an average tile image for each color channel from all tiles in the current section. An example average tile is shown in Figure 4. Though it is possible to generate a standard average tile image for use across different brain volumes, it will not be as effective in correcting the lens vignetting effects. Individual differences in intensities between scanned brains or different scan settings require the average tiles to be computed for each brain.

We define these average tile images as profiles. This step results in four profiles in total, one for each color channel. Though activated cells will present higher intensity values than the surrounding tissue, corresponding tiles in different sections help average these values to keep the profile consistent. Next, we used these profiles to adjust the brightness of each tile pixel-by-pixel.

**Figure 5:**
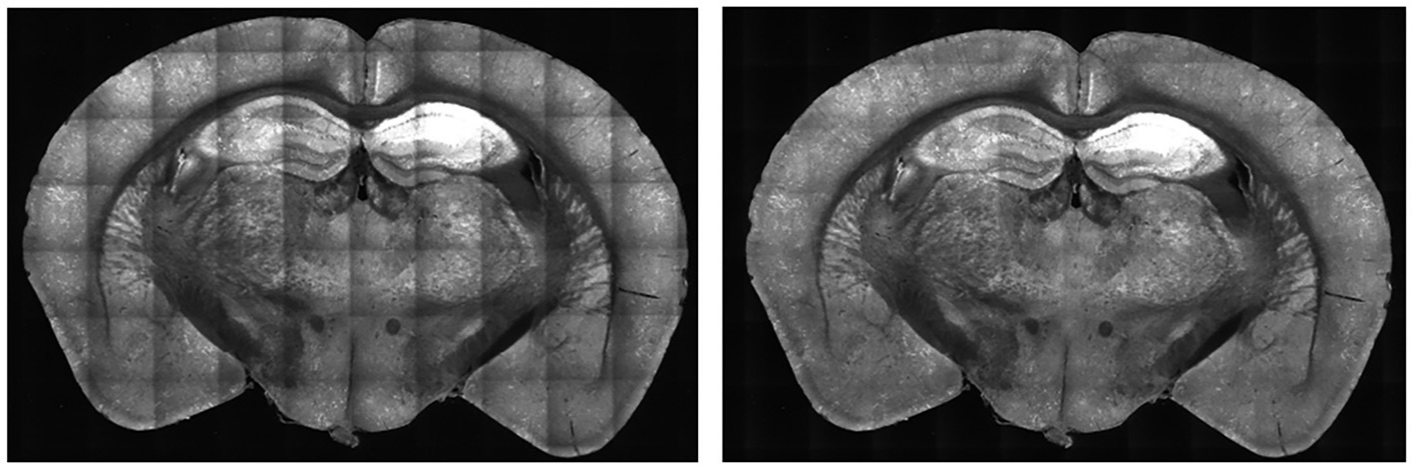
Comparison of B0039 images before **(left)** and after **(right)** applying brightness normalization. Before the step, the edges are darker than the center. After brightness normalization, this discrepancy is significantly reduced.

For each tile image, we adjusted pixel values according to its profile to normalize the brightness across the tile. This process normalizes the pixel values into a scale with values ranging between 0 to 1. For each pixel of a tile image, we divided it by its corresponding pixel in the profile. For darker pixels, this process increased their value, while conversely, it reduced the brightness of overly illuminated pixels. This step ensured that the final image presented a balanced and uniform appearance, effectively mitigating the problem of overly dark and bright spots in the stitched 2D section. Some minor tiling effects may still be present in the output, but we have not found these inhomogeneities to adversely affect the image-processing algorithms or quantification in our pipeline. As shown in Figure 5, there was an enhancement in image clarity before and after applying brightness normalization.

##### 2.4.2.2 Deformation correction

Lens distortion is a common problem on many imaging platforms and can cause issues with discontinuities in stitching processes due to nonlinear deformation. This distortion effect becomes apparent when using a lens to image an electron microscopy (EM) grid consisting of orthogonal lines. Correcting these distortions is critical, as it directly impacts the fidelity of the 2D section images. Doing so ensured that the tiles accurately represented the brain's actual structure. This distortion effect could be corrected by determining the extent of deformation and applying transformations on the tiles to correct for lens distortion.

**Figure 6:**
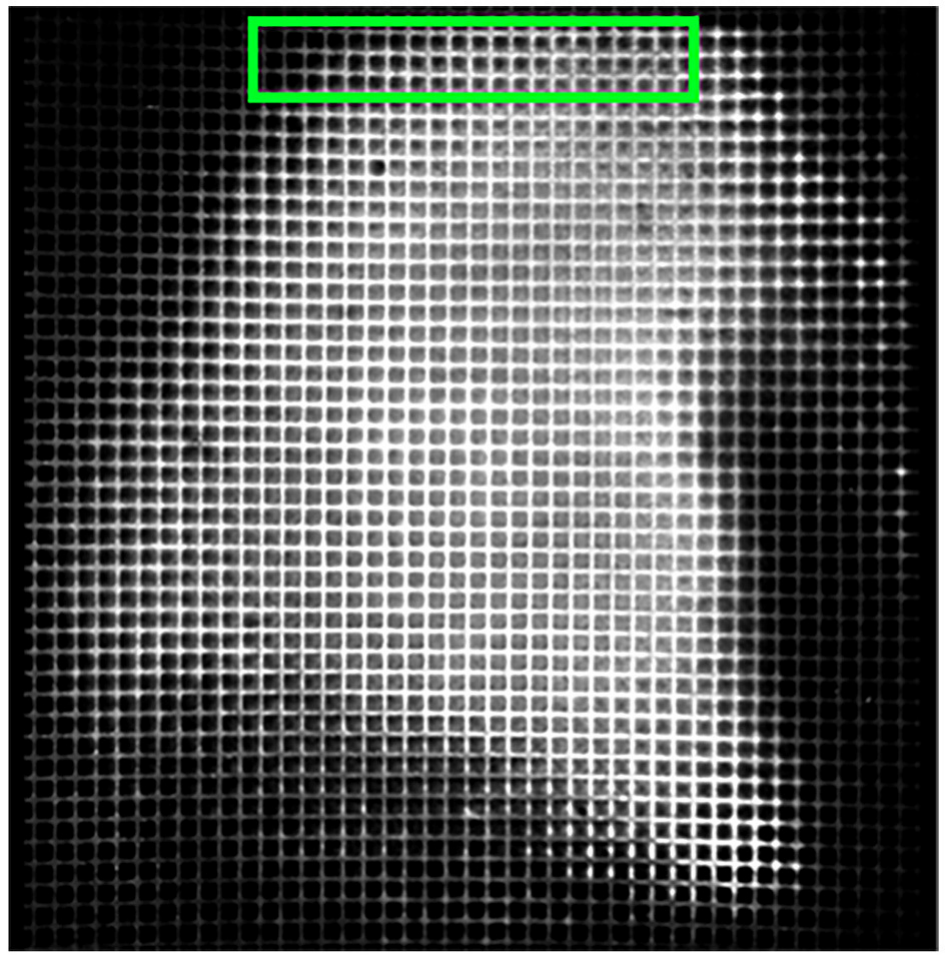
16 × objective lens imaged 1,000 mesh EM grid. Noticeable lens distortion manifests at the top and bottom of both images, with some cells along the edges being incomplete, or cut off along the edges. Taking the area within the green box as an example, the horizontal grid lines appear bent compared to the straight top and bottom edges of the green box.

We first employed 16 × objective lenses to capture images of a 1,000 mesh EM grid, featuring a 25 μm pitch, 19 μm hole size, and 6 μm bar width. Distortions were particularly noticeable along the tile edges, as depicted in Figure 6, and manifested as unnatural curvature or skewing of the grid lines, which should otherwise appear orthogonal.

Our approach to adjusting this deformation began with analyzing an EM grid, which served as a ground truth to help correct lens distortion. By comparing the deformation between a ground truth EM grid and a corresponding imaged EM grid output from TissueCyte, we can determine the deformation parameters to transform the output to match the ground truth template. Upon imaging the EM grid, the edges of this grid image often showed partial cells due to incomplete capture or lens distortion, as presented in Figure 6. These partial cells contained insufficient information, making them inapplicable for further analysis. To overcome this, our algorithm automatically identified the four corner points for each whole cell on the grid. These points mark the boundaries of the fully complete cells. Identifying these corner points was vital as they served as anchors for aligning the distorted grid with the template.

To aid in the correction process, we created a template grid with equally spaced horizontal and vertical lines, designed to represent an undistorted version of the EM grid. The number of lines in both directions corresponded to the number of complete cells identified within the corner points of the distorted grid. The corner points in the template grid were easily identified, as the lines were equally spaced.

We then applied a computer vision technique called homography transformation (Luo et al., 2023) to adjust the original grid to ensure it matches the template grid's dimensions and layout. This is done by using the sets of four marked corners previously identified on the grid and by establishing correspondences between those points and the equivalent points on the template. A projective transformation is then applied to the grid.

**Figure 7:**
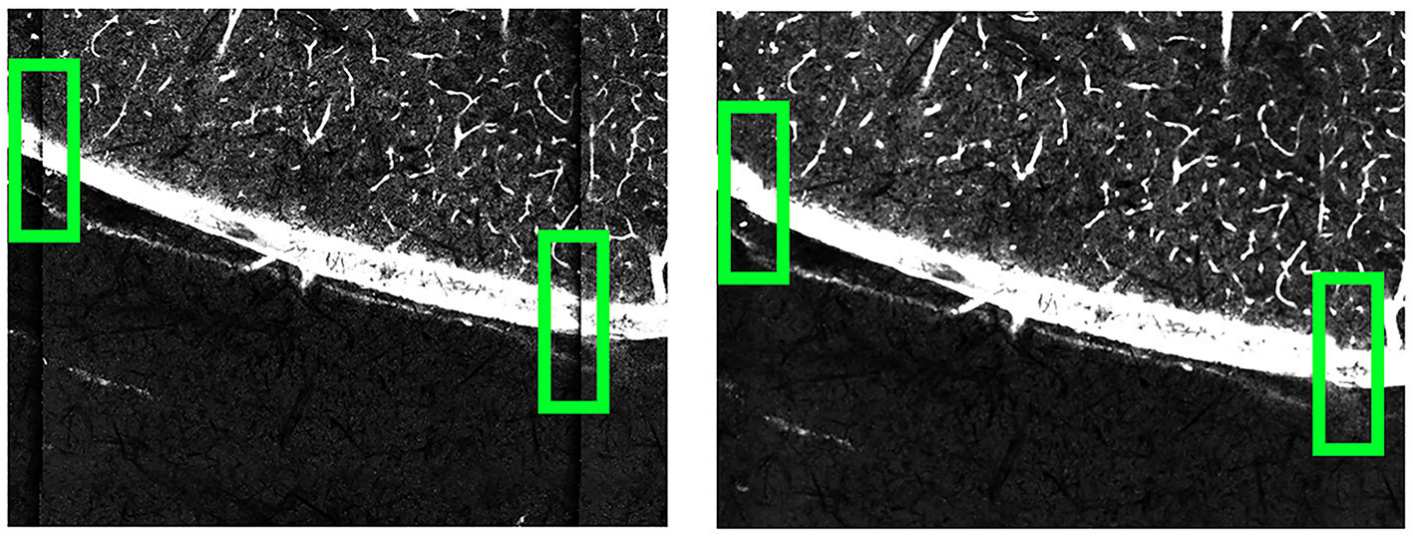
Comparison of the B0003 brain images before **(left)** and after **(right)** applying deformation correction. Before the correction, the tile edges do not align seamlessly with neighboring tiles. After the correction, the connections between neighboring tiles become smoother.

Once the grid is transformed to match the template, we fit Bezier surface patches (Goshtasby, 1989) to the grids using each set of four points. Each point within a Bezier patch can used as control vertices and matched with points in the other grid to establish correspondences. A bicubic spline function (De Boor and De Boor, 1978) was used to model the deformation between these correspondences. By displacing the control vertices to match their corresponding vertices in the template, each pixel within the deformed grid was mapped to its new location by interpolating between corresponding points. This pixel-by-pixel adjustment successfully corrected the deformation, resulting in an image significantly less influenced by lens-induced distortions. As these deformations are consistent and uniform across different sections and brains, these deformation parameters do not need to be recomputed for each new brain dataset; these parameters can be reused for different scans on the same imaging machine. We show how the deformation correction process affects our images in Figure 7.

#### 2.4.3 Converting 2D images into a 3D data

To convert 2D section images to a coherent 3D structure, we stacked each of the 2D sections to create a 3D array. Each section was reassembled based on their sequential order to reconstruct the full three-dimensional anatomy of the brain. This 3D array was then compressed and saved into the Neuroimaging Informatics Technology Initiative (NIfTI) format. Multiple NIfTI files were saved into an output folder containing the full-resolution images and down-sampled versions to match the Allen CCF format. These section images were efficiently compressed and stored, minimizing required storage space while maintaining quick data access.

### 2.5 Utilizing Texera for collaborative data analytics using workflows

**Figure 8:**
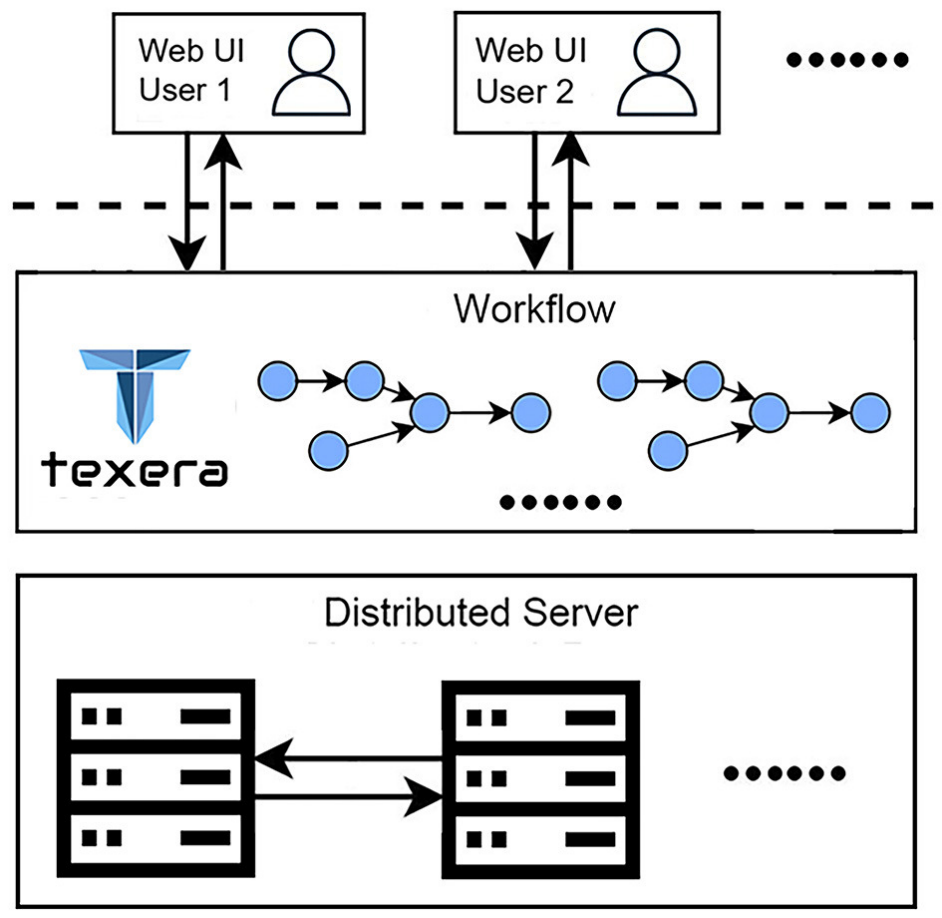
Overview of Texera, a collaborative data analytical workflow system with a distributed execution engine. The platform allows multiple users to access and modify workflows simultaneously.

This work highlights two critical requirements for our data-analysis process. First, the work involves a collaborative effort from three research teams specializing in neuroscience, computer vision, and data systems. The diverse expertise and skill sets of the teams necessitate a platform that supports seamless collaboration. Second, processing a large amount of brain image data (172,480 files and 224 GB per brain) requires an efficient solution to reduce the time. To meet these requirements, we utilize Texera (Texera, 2024), a platform to support collaborative data analytics using workflows. Texera provides a web-based cloud service for data analytics and allows users to analyze data without installing software on their computers. It is a collaborative environment similar to existing collaboration services such as Google Docs and Overleaf, allowing users from diverse disciplines to jointly edit workflows and manage their executions. Texera uses a distributed computation engine that can allocate its workload across a cluster of machines. This capability reduces the processing time on large volumes of data. We build workflows on Texera to conduct image pre-processing, 2D–3D conversion, and 3D visualization of brain data. An overview of Texera is presented in Figure 8.

Here, we present an experience of how users, Alice and Bob, collaborate using Texera. Alice first logs into Texera and creates a new workflow. Then she shares it with Bob. After that, she adds a new operator to the canvas and starts to work on it. When Bob joins the workflow, he sees the operator Alice is working on. If Bob decides to add a second operator, he places it on the canvas and links it to the first one. After Alice finishes her changes, the two users work on the second operator together. Once they are done, Bob clicks the “Run” button to execute the workflow.

In addition to supporting over 100 operators for data processing, machine learning, and visualization, Texera also supports user-defined operators in several popular programming languages, including Python, Java, and R. This capability allows users to define their own custom operators in these languages based on different needs and their programming skills.

**Figure 9:**
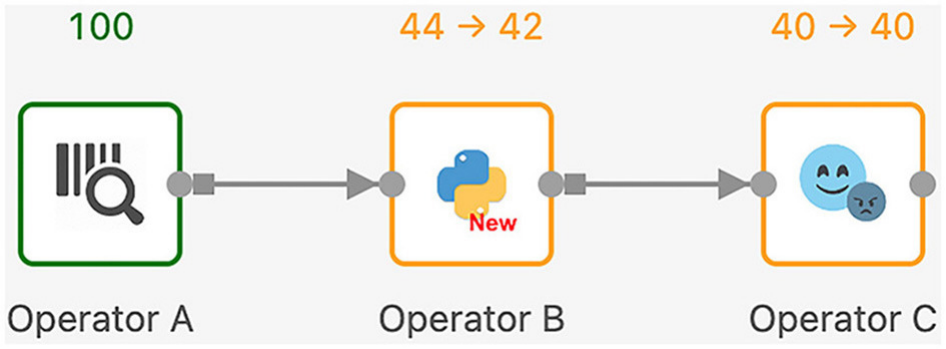
A Texera workflow that consists of three operators *A*, *B*, and *C*. The data flows from operator *A* to operator *B* and eventually to operator *C*. Each operator shows its number of input tuples and number of output tuples. Green operators have completed their execution, while yellow operators performing their computations.

Next, we discuss the execution model of Texera with an example workflow shown in Figure 9. In Texera, an operator serves as the minimal unit of data transformation. It receives input data, performs a transformation, and outputs results. A directed edge between operators indicates the direction of data flow. In Figure 9, operator *A* sends its output data to operator *B*, which sends its output data to operator *C*. By connecting operators with directed edges, we construct a directed acyclic graph (DAG) as a workflow. Texera processes data tuples using a pipelining approach, where multiple operators can process data concurrently. Each operator can be executed using multiple workers.

## 3 Results

In this section, we report our experimental results.

### 3.1 Texera workflows

We constructed several Texera workflows following the methods described in Section 2.4, including a workflow for tile adjustment, stitching, and 2D-to-3D conversion. Next, we present details of these workflows.

#### 3.1.1 Workflow 1: tile adjustment and stitching

**Figure 10:**
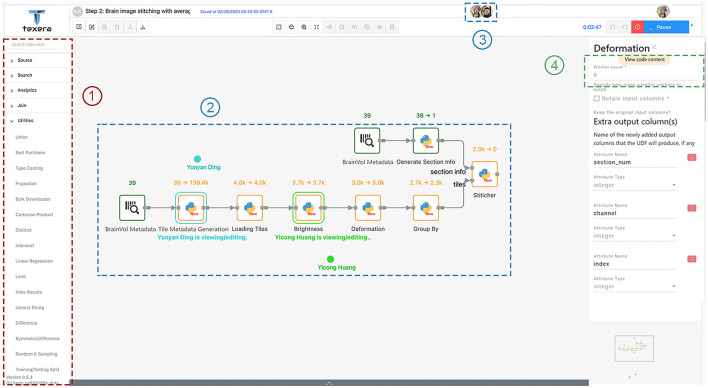
Workflow 1 for tile adjusting and stitching, with a few key components in dotted rectangles: (1) operators for users to drag and drop into the canvas; (2) shared editing that allows multiple users to view and edit the same workflow simultaneously; (3) icons of concurrent users working on the workflow; (4) the number of workers for each operator.

This workflow performs the stitching process mentioned in Section 2.4.1 and improves the tile image quality as mentioned in Section 2.4.2. It converts the raw tile images obtained from TissueCyte to coherent, high-quality 2D section images. As shown in Figure 10, this workflow has the following operators.

1. Loading brain volume metadata: This operator, denoted as “BrainVol metadata” in the workflow, is used to upload the text file that contains the brain volume metadata and process the content.
2. Generating tile metadata: This operator takes the brain volume metadata as input and generates the metadata for each tile that includes the file path, section range, boundary position, margin value, size information, channel, and labeled section index.
3. Generating section information: Similar to the previous operator, this operator takes the brain volume metadata as input and computes the required metadata information for stitching, such as brain section image dimension, color channel numbers, and brain ID.
4. Loading tiles: This operator uses the provided tile metadata and loads corresponding tile images from the given file paths in the tile metadata. During this process, it checks for any missing tiles and tags them with a boolean flag used by the subsequent operators to decide whether they should substitute the tile with a placeholder zero image, where all pixel values are set to 0.
5. Brightness normalization: This operator adjusts the brightness level of the tile images. It loads the average profiles for the four color channels generated from another workflow. For each tile image, the corresponding color channel's profile is applied to do the adjustment. Within each tile image, pixel values are modified according to the approach outlined in Section 2.4.2
6. Deformation correction: This operator performs the step as described in Section 2.4.2. In the initial computation, we save the necessary parameters to correct lens deformation. In subsequent runs, we apply these transformation parameters to the tile images and ensure accurate correction of lens distortions. The output image tiles then are ready for stitching.
7. Categorization: This step sorts tiles based on their section number and ensures the correct identification and grouping of tiles. It distributes the sorted tiles to a worker responsible for stitching the particular section.
8. Stitching: This operator stitches tile images within the same section into a single-section image as defined in Section 2.4.1. It creates a canvas with the width and height as specified by the section metadata. It associates each input tile with a specific coordinate indicating its relative position in the section. It positions them to the correct position pixel-by-pixel based on input parameters, which ensures that the overlapping positions between neighboring tiles are resolved without trimming the overlapping regions. This operator takes the coordinate and translates the tile to the exact position on the canvas. After filling the canvas, it outputs a new image representing one complete brain section.

The final compiled images are converted to the NIfTI file format, resulting in a comprehensive volumetric representation of the entire brain. This representation consists of ~280–300 stitched brain sections, each of which has 8, 716 × 11, 236 pixels, with a pixel-to-pixel spacing of 1.25 μm and a section-to-section spacing of 50 μm.

#### 3.1.2 Workflow 2: 2D to 3D conversion and 3D visualization

**Figure 11:**
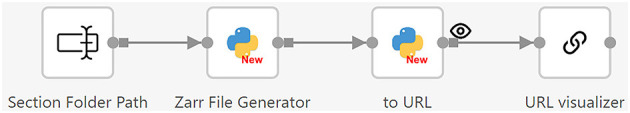
Workflow 2: converting 2D section images to a 3D model for visualization. It reads the folder path of the section images, converts the images to Zarr-format files, and generates URLs for Neuroglancer to visualize.

After all section images are stitched, we use the workflow shown in Figure 11 to convert them to a 3D model for visualization. This workflow consists of two steps. First, the *Zarr File Generator* operator receives the folder path of all the section images, loads the section images, and generates Zarr image files (Open Geospatial Consortium, 2023). Zarr is a format for storing large multi-dimensional arrays. It organizes data into hierarchical groups, where each level has datasets with arbitrary JSON metadata files (Moore and Kunis, 2023). The Zarr format supports fast processing and easy access, making it particularly useful for handling large volumes of dense, multi-dimensional arrays, such as brain image data. Since individual files within a Zarr dataset are accessible via predefined paths, they can be easily accessed using a Web browser (Moore and Kunis, 2023). We utilize a web-based tool called Neuroglancer (Maitin-Shepard et al., 2021) to visualize the 3D model. To allow users to easily do the visualization using a Web browser, we integrate Neuroglancer into Texera.

This workflow has three operators specifically designed to convert 2D section images into 3D data, and then to visualize it as a 3D model.

- Zarr file generation: This operator takes the folder path of the section images as input and loads the section images one by one based on their section numbers. It scales the section images and adjusts the exposure to increase image readability. After that, it reads the section images into a Dask array (Rocklin, 2015). The Dask format is a specific adaptation of the Zarr format, designed to meet the needs of complex biological imaging data (Moore et al., 2023). The operator uses these Dask arrays to write all the section images to an OME-Zarr format onto the disk. Then, it outputs the folder path where the Zarr files are stored.
- 3D model visualizer: This operator accepts the Zarr file folder as input, converts the folder into a Neuroglancer URL, and sends it to the URL visualization operator to show the 3D brain model.

### 3.2 Optimizations

Processing large amounts of data for each brain is time-consuming. In this section, we discuss a few optimization techniques we used on Texera to reduce the processing times. For all the results in this section, we ran the workflows on a 32-core CPU and 128 GB RAM machine with the Ubuntu Linux 20.04 operating system. Initially, we combined all the tasks as a single operator. In this approach, we processed the tile images one by one and loaded all tile images for each section to perform stitching, and the entire process took around *15* h to finish. This method of pre-processing brain images was notably time-consuming. To accelerate this process, we explored several optimizations.

#### 3.2.1 Using multiple operators

An operator doing too much computation can be hard to optimize. Splitting the operator into smaller, specialized components allows for fine-grained tuning and optimizations. Thus we decomposed the operator into separate operators, each of which performs a specific task. The operators are shown in Figure 10.

#### 3.2.2 Enabling parallelism in each operator

The capability to process each tile independently presents an opportunity for parallel processing. We mark those operators that can be parallelized so that Texera can facilitate data shuffling and partitioning to ensure that each worker receives and processes a portion of the input data.

#### 3.2.3 Maximizing pipelining between operators

We noticed that operators affect each other's execution time. Specifically, in a pipelining setup, the slowest operator can slow down the execution of the entire workflow. To speed up the process, we focused on making the slowest, or bottleneck, operators faster by giving them more workers. We carefully chose how many workers to assign to each operator based on their speed. The goal was to balance the workflow so that over a certain period, all operators would process data at a similar pace. This means that as soon as a worker finished processing one piece of data, another piece was ready to be processed, minimizing the idle time of the worker. We found that the deformation correction and stitching operators were the main bottlenecks. To address this issue, we allocated six workers to the deformation correction operator and four to the stitching operator. As shown in the diagram in Figure 10, the tile-loading operator had two workers, while all other operators in Workflow 1 had one worker each.

For a more comprehensive comparison, we also implemented an approach using traditional Python scripts, optimizing parallelism with joblib^1^. This method allowed tasks to be executed simultaneously across multiple cores on a single machine. We evaluated three approaches: *Traditional Python scripts, Python script with joblib*, and *Texera workflows with optimizations*. All were tested on the brain B0039 using the same machine mentioned earlier, with aligned Python and library versions. We compare these methods in terms of execution time and user workload, as detailed below:

- Traditional Python scripts: To execute the Python scripts, users needed to use the command line and provide the tile image path. This method took 15 h to complete the entire pre-processing and stitching tasks.
- Python script with joblib: Similar to the traditional method, users executed the script via a command line and provided the tile image path. This approach reduced the completion time to 3.5 h.
- Texera workflows with optimizations: To execute the workflow, users first modified the “source text input” operator with the folder directory and then click the “Run” button. The workflow completed the entire pipeline in 2.5 h.

The Texera approach showed a significant improvement over the initial run using traditional Python scripts, achieving an 83.33% reduction in the processing time. Compared to the joblib optimization approach, the optimized Texera workflow resulted in a further 28.57% reduction in the processing time.

### 3.3 Visualization results

**Figure 12:**
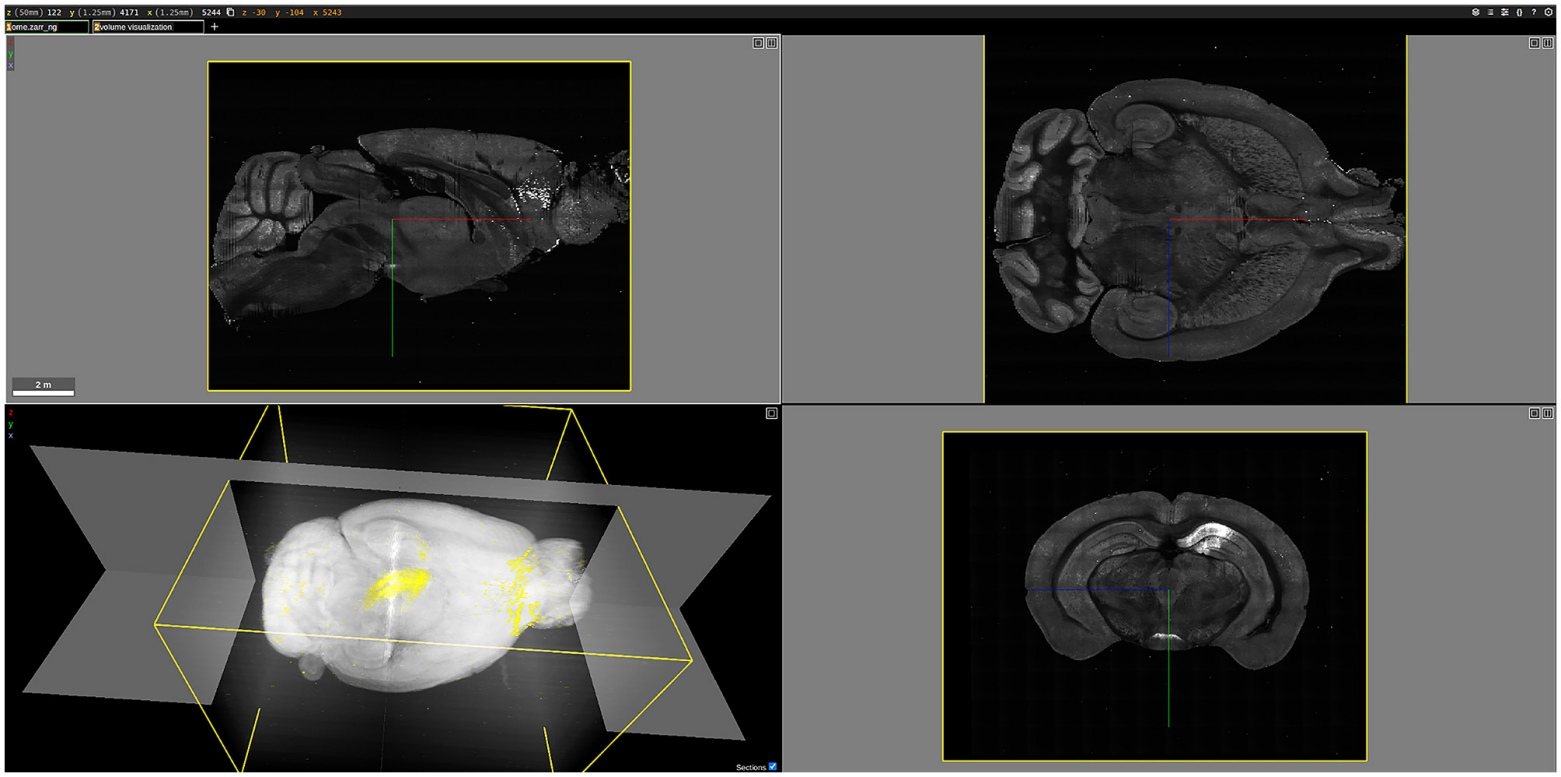
3D visualization of the brain labeled B0039, rendered using Neuroglancer. The **(bottom-left)** figure shows a 3D volume visualization, highlighting the fluorescently labeled cells in yellow against a white background. The other three figures illustrate different planes of the brain: the **(top-left)** figure shows the sagittal view, the **(top-right)** figure shows the horizontal view, and the **(bottom-right)** figure shows the coronal view, with bright areas indicating fluorescently labeled cells.

As an example, Figure 12 shows a three-dimensional (3D) visualization of the brain labeled B0039. For this brain, viral tracers were injected into the dCA1 region, resulting in the fluorescent labeling of all input neurons to the injection site. Once rendered in 3D, the brain could be examined in various two-dimensional planes. For instance, the bottom-right image in Figure 12 displays a coronal plane of the brain, where the brighter regions indicate fluorescently labeled cells. In the 3D visualization at the bottom left of Figure 12, the white areas represent tissue autofluorescence, while the yellow regions highlight the labeled cells.

## 4 Discussion

### 4.1 Collaboration on Texera

This work is a joint effort of three research teams from three disciplines: neuroscience, computer vision, and data systems. The neuroscience team provides the data, i.e., tile images, and standards for the output model accuracy. The computer vision team develops algorithms and techniques for processing images and 3D models. The data systems team is responsible for developing the Texera platform, leading the effort to construct workflows, and optimizing their execution performance.

In contrast to traditional methods of collaboration, which often involve cumbersome back-and-forth transfers of data or code, Texera provides a shared data science experience. Similar to Overleaf (Overleaf, 2024) for LATEX and Google Docs for rich text documents, Texera allows multiple collaborators to work on a workflow at the same time. The three teams use Texera to collaboratively edit a workflow, run it, and share its results. Take Workflow 1 in Section 3.1 as an example. The computer vision team works on the brightness and deformation operators, while the data systems team works on generating section metadata and loading tiles. During the execution of the workflow, each team can specialize in assessing the output of their respective operators and apply their expertise to ensure the operators' accuracy and correctness. The computer vision team, for example, can evaluate the efficacy of the brightness normalization operator by examining pixel generation, whereas the neuroscience team can review the visualization of the final 3D model to ascertain if it meets the desired accuracy and resolution standards.

### 4.2 Flexibility and generalizability

Our presented pipeline can be reproduced, extended, and reused to analyze other image data by researchers in the neuroscience community. This capability is crucial for ensuring scientific reproducibility and facilitating cross-validation. Additionally, the pipeline's adaptability allows for adjustments to accommodate more detailed image analysis with potentially larger data volumes. Workflows can be scaled efficiently to run on a cluster of machines to handle increasing data volumes, and optimizations can be tailored to meet diverse user requirements. Because of the modularity of workflow operators, many operators such as brightness normalization and deformation correction can be reused to process other image data. This functionality allows collaborators to share and reuse operators across different workflows.

### 4.3 Experiments on other systems

We have also compared Texera with two other popular workflow systems used in data analytics and machine learning: RapidMiner (Hofmann and Klinkenberg, 2016) and KNIME (Berthold et al., 2009). RapidMiner provides an integrated environment for data preparation, machine learning, and deep learning. KNIME facilitates a visual construction and interactive execution of data pipelines. It supports easy integration of data manipulation and visualization through the addition of new modules or nodes, where these nodes or modules are comparable to operators in Texera. Next, we compare them from three aspects

- Collaboration functionality: RapidMiner and KNIME allow workflow sharing through manual updates. Texera supports real-time collaboration, enabling multiple users to work synchronously on the same workflow.
- Execution models: RapidMiner and KNIME primarily support processing tabular data between operators. They execute their workflows operator by operator. Texera allows data to be processed on a tuple-by-tuple basis and supports pipelined execution, allowing multiple operators to process data concurrently.
- Deployment methods: RapidMiner and KNIME require users to install their software for usage. Texera offers a cloud-based service so that users can access their workflow using a web browser.

To further illustrate the performance differences, we constructed the tile adjustment and stitching workflow in RapidMiner with an identical structure to the one in Texera and tested them under the same conditions. Unfortunately, RapidMiner was unable to complete when processing more than eight sections of tile images. In another test involving the processing of a single section of tile images, we executed the workflows in both Texera and RapidMiner on the same computer machine. Each operator in the Texera workflow was assigned one worker. RapidMiner took 7 min and 16 s to complete the task, whereas Texera finished the same task in 4 min and 39 s.

This performance difference could be attributed to the inherent differences in their execution models. In our pre-processing steps for tile images, each tile is independent from each other. For example, while tile image *A* undergoes brightness normalization, tile image *B* can undergo deformation correction simultaneously. RapidMiner and KNIME utilize a parallel execution model known as data parallelism. Texera enables pipelined execution in addition to data parallelism, which aligns more closely with the inherent characteristics of this task. Consequently, for our task, Texera's parallel execution model utilizes CPU resources more effectively.

### 4.4 Related work

In the realm of imaging and 3D reconstruction of the entire mouse brain, two new methods prevail. Our study utilizes automated block-face serial imaging methods, including Serial Two-Photon Tomography (STPT/TissueCyte) (Ragan et al., 2012; Osten and Margrie, 2013; Kim et al., 2015), Fluorescence Micro-Optical Sectioning Tomography (fMOST) (Gong et al., 2013), and Block-face Serial Microscopy Tomography (FAST) (Seiriki et al., 2017, 2019). These methods integrate imaging with automated sectioning, facilitating efficient data acquisition. Alternatively, the second prevalent approach involves light-sheet microscopy of cleared mouse brain samples (Dodt et al., 2007; Chung et al., 2013; Renier et al., 2014; Jing et al., 2018; Susaki et al., 2020; Ueda et al., 2020; Kosmidis et al., 2021). We will compare the pros and cons of both methods across three dimensions: tissue preparation, imaging, and post-imaging processing.

For block-face serial imaging methods, tissue preparation involves postfixing brain samples in 4% paraformaldehyde (PFA) post-transcardial perfusion and embedding them in 3%–5% agarose, which typically requires about one day. Some protocols might include additional steps to enhance tissue stiffness, like soaking in acrylamide or sodium borohydrate, extending the preparation to two additional days. This method, taking ~2–3 days in total, preserves the brain's original morphology as no harsh chemicals are applied. Imaging a mouse brain using a TissueCyte microscope typically requires about 22 h to obtain four-channel images with a resolution of 1.25 μm in the *xy*-plane and 50 μm in the *z*-axis. Recent advancements, like FAST, can image the whole brain in 2.4–10 h with a resolution of 0.7 μm in the *xy*-plane and 5 μm in the *z*-sampling interval (Seiriki et al., 2019). High-resolution images are obtained, clearly depicting cellular structures and processes. The preservation of normal brain morphology simplifies the registration and other post-imaging processes, like automatic cell detection.

In contrast, light-sheet imaging of cleared samples involves a series of preparation steps including fixation, decalcification, decolorization, delipidation, and refractive index (RI) matching, typically requiring about a week (Dodt et al., 2007; Chung et al., 2013; Jing et al., 2018; Susaki et al., 2020; Ueda et al., 2020; Kosmidis et al., 2021). Methods using organic solvents may quench fluorescent signals, necessitating immunostaining, which can extend the entire process to ~1 month (Renier et al., 2014; Gao et al., 2024). The use of harsh chemicals often alters brain morphology, causing anisotropic expansion or shrinkage that complicates registration with standard brain templates like Allen's CCF. Although light-sheet microscopy can rapidly image cleared samples in about 2–3 h per brain, the spatial resolution might be compromised by imperfect clearing. Variations in refractive index within different brain regions can introduce optical aberrations, affecting image quality. Despite these challenges, ongoing technological improvements have enhanced the quality of axonal projection imaging via light-sheet microscopy (Tomer et al., 2014; Susaki et al., 2020).

In conclusion, while light-sheet microscopy offers rapid imaging of cleared brains, block-face serial imaging methods provide superior resolution and simpler post-processing due to better preservation of native brain morphology.

One novel aspect of our study lies in the adoption of Texera, which distinguishes itself as an ideal tool for our purposes. Its user-friendly interface, combined with an efficient pipeline structure, makes it a good choice for our image assembly needs. Other workflow systems such as Knime (Berthold et al., 2009) and RapidMiner (Hofmann and Klinkenberg, 2016) lack features that support real-time collaboration among users and their execution models are not well aligned with our work, as discussed in Section 4.3. Big data systems such as Spark (Zaharia et al., 2010) and Flink (Carbone et al., 2015) are for large-scale data processing, yet they lack collaboration features and a workflow interface needed by users with limited programming skills. There are cloud-based platforms that focus on biomedical research, such as Cavatica (Cavatica, 2024), which specializes in genomic data, and Galaxy (Afgan et al., 2018). Both platforms do not support real-time collaboration functionality, pipelined execution, nor computing on multiple machines. Texera has been selected as the ideal platform to fulfill our design and as a collaborative open-source alternative.

For sections of 2D biomedical image data, it is a common practice to stack these sections into a 3D array to create a volume. There exist multiple methods that can represent and render 3D data (O'Donoghue et al., 2018; Zhou et al., 2022). One method is based on volume or direct rendering, and it allows users to view entire datasets at once. This method utilizes ray casting and transfer functions, layers transparency through voxels, colors regions based on scalar values within the volume, and provides an accurate visualization of internal features. While direct rendering offers detailed insights into biomedical images, especially those with noise, it allows the visualization of features obscured by surface techniques, and it is typically slower and less suited for real-time applications. This is in contrast to surface rendering, which is fast, but does not store information past the modeled surface boundary (Kuszyk et al., 1996). For this reason, we use volume rendering.

### 4.5 Future works

In our current work, it is important to note that although the tile images significantly improve the raw images, they are not entirely free from the effects of lens distortion. Despite this, our existing correction method is sufficient for our subsequent analyses, including cell counting and registration. One research direction is to create a more robust solution to correct deformation to better preserve features at a vascular level so that we can enhance visualization results while still maintaining accuracy.

## 5 Conclusions

In this paper we present a novel pipeline that transforms mouse brain samples to detailed 3D brain models by using a collaborative data analytics platform called “Texera.” Our pipeline utilizes the tile images from a serial two-Photon tomography/TissueCyte system, then stitches tile images into brain section images, and constructs 3D whole-brain image datasets. The resulting 3D data supports downstream analyses, including 3D whole-brain registration, atlas-based segmentation, cell counting, and high-resolution volumetric visualization. Our work significantly accelerates research output and analyses, enabling faster and more detailed exploration of brain structures.

## Data availability statement

The raw data supporting the conclusions of this article will be made available by the authors, without undue reservation.

## Ethics statement

The animal study was approved by the University of California, Irvine Institutional Animal Care and Use Committee (IACUC, protocol #: AUP-22-163) and the Institutional Biosafety Committee (IBC). The study was conducted in accordance with the local legislation and institutional requirements.

## Author contributions

YD: Writing – review & editing, Writing – original draft. YH: Writing – review & editing, Writing – original draft. PG: Writing – review & editing. AT: Writing – review & editing. AC: Writing – review & editing. MG: Writing – review & editing. XX: Writing – review & editing. CL: Writing – review & editing.

## Footnotes

^1^https://joblib.readthedocs.io/ (accessed February 28, 2024).
